# Supplementary material for: Analysis of PPARγ Signaling Activity in Psoriasis
Source: Int J Mol Sci. 2021 Aug 10;22(16):8603. doi: 10.3390/ijms22168603 (PMC8395241; doi:10.3390/ijms22168603)
Supplement: Supplementary file 1 [file ijms-22-08603-s001.zip › Supplemental materials_Analysis of PPARg signaling activity in psoriasis/Pathway models/Models images and html files/Model of PPARG signaling in psoriasis/179381.html]

ROS


# Small Molecule ROS

|  |  |
| --- | --- |
| URN | urn:agi-smol:ROS |
| Total Entities | 0 |
| Connectivity | 13892 |
| Name | ROS |
| Class | Endogenous compound |

---

|  |  |
| --- | --- |
| Pathway | Alzheimer's Disease |
|  | Skin Aging |
|  | Apoptosis of Cochlear Hair Cells (Noise-Induced, Mouse Model) |
|  | Oncogene Induced Cellular Senescence |
|  | Melatonin Effects on Circadian Cycle |
|  | SIRT6 Signaling in Aging |
|  | SIRT1 Signaling in Aging |
|  | Fatty Acids Cause Oxidative Stress and Mitochondria Decline in Aging |
|  | Protein Oxidation and Aggregation in Aging |
|  | Extracellular Matrix Reformation in Aging |
|  | NOD-like Receptors |
|  | Scavenger Receptors in Platelet Activation |
|  | Toll-like Receptor Independent Sterile Inflammation |
|  | Necrosis |
|  | Lipoxin A4/FPR2-Related Neutrophil Depression |
|  | Eicosanoids in Inflammation |
|  | Neutrophil Activation via LTB4/Polyisoprenyl Phosphate Signaling |
|  | Eosinophil Activation |
|  | Macrophage M1 Lineage |
|  | CD16/CD14 Proinflammatory Monocyte Activation |
|  | Neutrophil Activation via FCGR3B |
|  | Neutrophil Degranulation via FPR1 Signaling |
|  | Neutrophil Chemotaxis |
|  | Neutrophil Activation via Adherence on Endothelial Cells |
|  | Melatonin in Cell Survival and Antioxidant Response |
|  | Scavenger Receptor OLR1 in Inflammation Related Endothelial Dysfunction |
|  | RAGE/AGER and S100 Proteins in Cardiovascular Injury |
|  | Dilated Cardiomyopathy |
|  | Endothelial Cell Dysfunction in Pulmonary Hypertension |
|  | Smooth Muscle Cell Dysfunction in Pulmonary Hypertension |
|  | Reperfusion Injury in Myocardial ischemia |
|  | Myocardial Infarction |
|  | Preconditioning Ischemia |
|  | Vascular Reactivity in Raynaud Disease |
|  | Cold Stress and Adrenoceptor alpha 2C Signaling in Raynaud Disease |
|  | NO Synthesis in Early Vasospasm |
|  | Hemoglobin Reduction and Leukocyte Adhesion Initiate Vasospasm |
|  | Sebocyte Proliferation in Acne Vulgaris |
|  | Androgens Synthesis in Sebocytes |
|  | Cell Cycle Dysregulation in Melanoma |
|  | Melanoma |
|  | MITF as a Regulator of Melanoma Cell Development |
|  | Extracellular Matrix Degradation in Skin Aging |
|  | Melanocyte Role in Skin Aging |
|  | Apoptotic Keratinocytes Clearance Recession in Systemic Lupus Erythematosus |
|  | Plasmacytoid Dendritic Cell in Diabetes Mellitus Type 1 |
|  | Toll-like Receptors in beta-Cell |
|  | Macrophage Function in Diabetes Mellitus Type 1 |
|  | Conventional Dendritic Cell Role in Diabetes Mellitus Type 1 |
|  | beta-Cell Death in Diabetes Mellitus Type 2 |
|  | Advanced Glycation End Products Pathway in Diabetic Microangiopathy |
|  | Polyol Pathway in Diabetic Microangiopathy |
|  | Peripheral Tissue Microangiopathy |
|  | Tubular Cell Dysfunction in Progressive Diabetic Nephropathy |
|  | Endothelial Cell Dysfunction in Early Diabetic Nephropathy |
|  | Oxidative Stress in Diabetic Neuropathy |
|  | AGE Related Insulin Resistance in Skeletal Muscles |
|  | Lipid Metabolism Impairement in non-Alcoholic Fatty Liver Disease |
|  | beta-Cell Recovery in Diabetes Mellitus |
|  | Hyperglycemia and Hyperlipidemia Trigger beta-Cell Apoptosis |
|  | FOXO1 and SREBP-1C Role in beta-Cell Suppression (Rodent Model) |
|  | Hashimoto's Thyroiditis |
|  | Hashimoto's Thyroiditis Triggers |
|  | Fatty Acids in Hypertension and Atherosclerosis Development |
|  | Macrophage Dysfunction |
|  | Eosinophil Survival in Asthma |
|  | Eosinophil Activation and Degranulation in Asthma |
|  | Airway Smooth Muscle Cell Contraction |
|  | Neutrophil Degranulation via FPR1/IL8 |
|  | CFTR Facilitates Oxidative Stress in Airway Epithelium in CF |
|  | Airway Epithelial Cell Dysfunction |
|  | Monocytes Function in Gout |
|  | Dendritic Cell Dysfunction in Psoriatic Arthritis |
|  | Skin Fibrosis |
|  | PAPA Syndrome |
|  | TNF Receptor Associated Periodic Syndrome |
|  | Cryopyrin (NLPR3) Associated Periodic Syndromes |
|  | Familial Cold Autoinflammatory Syndrome 2 |
|  | Familial Mediterranean Fever |
|  | Glutamate Toxiicity Effects on Striatum Medium Spiny Neurons in Hantington's Disease |
|  | IL6 Overexpression After Contraction in Skeletal Muscles in Muscular Dystrophies |
|  | Muscular Dystrophies |
|  | Dystrophin Glycoprotein Complex Signaling in Muscular Dystrophies |
|  | Ca2+ Toxicity in Neurons |
|  | Ketogenic Diet Mechanism (Hypothesis) |
|  | PARK7 and LRRK2 in Young Onset Parkinson's Disease |
|  | Neurotoxin Effects in Parkinson's Disease |
|  | SNCA in Young Onset Parkinson's Disease |
|  | Glutamate Mediated Excitotoxicity in Amyotrophic Lateral Sclerosis |
|  | Oxidative Stress, All-Trans-Retinal and Lipofuscin Toxicity in AMD |
|  | Ca2+ Toxicity in Lens Cells |
|  | Diabetes Induced Cataract |
|  | Age-Related Cataract |
|  | Cataract, Congenital |
|  | Aminoglycosides and Cisplatin Induced Ototoxicity (Mouse Model) |
|  | Immune System Role in Otitis Media |
|  | Alveolar Macrophages Dysfunction in COPD |
|  | Neutrophil Associated Lung Damage in Chronic Obstructive Pulmonary Disease |
|  | CAV1 Dependent Senescence of Lung Fibroblasts |
|  | VEGFA High Expression Level in Pulmonary Emphysema |
|  | T-Cell Cytotoxic Activation |
|  | Mucin Hyperproduction in Goblet and Mucous Cells |
|  | Penile Detumescense Augmentation Mechanisms |
|  | Advanced Glycation End Products in Diabetes or Age Related Erectile Dysfunction |
|  | Ammonia Effects on Brain Cells |
|  | CD157/ITGB2 Signaling in Myeloid Cell |
|  | Acetaminophen Induced Hepatotoxicity |
|  | Asbestosis- and Silicosis-Associated Inflammation Provokes Cancer Development |
|  | Oxidized LDL/OLR1 Activates Cell Proliferation in Cancer |
|  | HIF1A as Master Regulator of Angiogenesis in Cancer |
|  | Invadopodia Formation in Cancer Cells |
|  | Respiratory Chain Impairment in Cancer |
|  | Metabolic Effects of Oncogenes and Tumor Suppressor in Cancer Cells |
|  | Glutamine in Cancer Metabolism |
|  | Tumor Infiltrating Macrophages in Cancer Progression and Immune Escape |
|  | Myeloid Derived Suppressor Cells in Cancer Immune Escape |
|  | HIF1 Signaling |
|  | ER Stress (Unfolded Protein Response) |
|  | Oxidative Stress Protection via AHR-NFE2L2-NQO1 Signaling in Keratinocytes |
|  | e |
|  | pathway 4 |
|  | sialic |
|  | Model of PPARG signaling in psoriasis |
|  | 2\_Hyperseborrhea in acne vulgaris |
|  | 2\_Oxidative stress, all-trans-retinal, and lipofuscin toxicity in agerelated macular degeneration |
|  | 2\_Diabetes-induced cataract |
|  | 3\_Congenital cataract |
|  | 1\_Age-related cataract |
|  | 1\_Low-density lipoprotein internalization and the accumulation of lipids in atherosclerosis |
|  | 3\_Arterial wall inflammation and calcification in atherosclerosis |
|  | 2\_The intense proliferation and contractile activity of smooth muscle cells in pulmonary hypertension |
|  | 1\_Cigarette smoke causes dysfunction and production of inflammatory mediators in alveolar epithelial cells |
|  | 3\_Role of alveolar macrophages in pulmonary tissue destruction in COPD |
|  | 2\_3\_Eosinophilia and neutrophilia in asthma: Neutrophil chemotaxis and activation |
|  | 4\_1\_Airway smooth muscle cells contraction |
|  | 1\_Antigen-presenting cells promote islet dysfunction and immune system activation in T1D |
|  | 1\_Microangiopathy in diabetic neuropathy |
|  | 1\_Glutamate and calcium excitotoxicity of motor neurons in ALS |
|  | Figure 4\_Diabetes mellitus Type 1: conventional dendritic cell |

---

|  |  |
| --- | --- |
| MedScan ID | 1810338 |

---

|  |  |
| --- | --- |
| Alias | free oxygen species |
|  | oxygen radicals |
|  | oxygen radical |
|  | reactive oxygen intermediates |
|  | ROS |
|  | free oxygen radical |
|  | oxygen free radicals |
|  | reactive oxygen species |
|  | oxygen free radical |
|  | free oxygen radicals |

---
